# Combining Baerveldt Implant with Trabectome Negates Tube Fenestration: A Coarsened-matched Comparison

**DOI:** 10.18502/jovr.v15i4.7789

**Published:** 2020-10-25

**Authors:** Hamed Esfandiari, Kiana Hassanpour, Peter Knowlton, Tarek Shazly, Mehdi Yaseri, Nils A. Loewen

**Affiliations:** ^1^Department of Ophthalmology, School of Medicine, University of Pittsburgh, Pittsburgh, Pennsylvania, United States; ^2^Ophthalmic Research Center, Research Institute for Ophthalmology and Vision Science, Shahid Beheshti University of Medical Sciences, Tehran, Iran; ^3^Department of Epidemiology and Biostatistics, School of Public Health, Tehran University of Medical Sciences, Tehran, Iran; ^4^Department of Ophthalmology, University of Würzburg, Würzburg, Germany

**Keywords:** Ab Interno Trabeculectomy, Baerveldt Glaucoma Implantation, Glaucoma Drainage Devices, Trabectome Surgery, Tube Ligation

## Abstract

**Purpose:**

To assess the efficacy and survival rate of the Trabectome-mediated ab interno trabeculectomy combined with non-fenestrated Baerveldt glaucoma implant compared with the Baerveldt glaucoma implant alone.

**Methods:**

In this retrospective comparative case series, 175 eyes undergoing primary glaucoma surgery (Baerveldt–Trabectome [BT] group: 60 eyes and Baerveldt [B] group: 115 eyes) were included. Participants were identified using the procedural terminology codes. Groups were then matched by Coarsened Exact Matching that resulted in the inclusion of 51 eyes in each group. The primary outcome measure was surgical success defined as 5 mmHg < intraocular pressure (IOP) ≤ 21 mmHg, and IOP reduction ≥ 20% from baseline, and no need to reoperation for glaucoma. Secondary outcome measures were IOP, number of glaucoma medications, and best-corrected visual acuity (BCVA).

**Results:**

The cumulative probability of success at one year was 61% in the BT group and 50% in the B group. IOP decreased from 23.5 ± 2.4 mmHg at baseline to 14.1 ± 2.7 mmHg at the final follow-up in the BT group (*P* = 0.001). The corresponding values for the B group were 23.2 ± 2.0 mmHg and 13.9 ± 1.6 mmHg, respectively (*P* = 0.001). There was no significant difference between the groups in terms of IOP at the final follow-up (*P* = 0.56). The number of medications at baseline was 2.3 ± 0.3 in both groups. However, the BT group needed fewer drops at all postoperative time intervals and used 1.1 ± 0.3 versus 2.0 ± 0.4 eye drops (group B) at the final follow-up visit (*P* = 0.004). Eyes in B with phacoemulsification had a significantly higher IOP on day 1 compared to B (23.2 ± 14.3 versus 17.9 ± 11.4, *P* = 0.041). During the one-year follow-up, 7 (13.7%) patients in BT group and 18 (35.2%) in B group experienced hypotony (*P* = 0.04). No dangerous hypotony or hypertension occurred in BT group. The mean BCVA at baseline was 0.64 ± 0.85 logMAR and changed to 0.55 ± 0.75 logMAR in BT and B groups, respectively (*P* = 0.663). The corresponding numbers for the final follow-up visit was 0.72 ± 1.07 and 0.63 ± 0.97 logMAR, respectively (*P* = 0.668).

**Conclusion:**

We observed similar rates of success and IOP reduction using BT and B techniques. BT group needed fewer glaucoma medications. Tube fenestration was unnecessary in BT group resulting in less postoperative ocular hypotony and hypertension. The results of our study indicate that additional trabectome procedure makes Baerveldt glaucoma implant safer, easier to handle, and more predictable in the most vulnerable patients with advanced glaucoma.

##  INTRODUCTION

Trabeculectomy and large glaucoma drainage devices (GDDs) are often chosen as primary surgical interventions for refractory glaucoma with a low target intraocular pressure (IOP). Both allow to bypass the impaired conventional outflow route and can achieve IOPs below that of the episcleral veins. Trabeculectomy can be associated with a high rate of failure and sight-threatening complications.^[1,2,3,4]^ Recent studies show GDDs have the same or a higher success rate than trabeculectomy in end-stage glaucoma but a better safety profile.^[1,2,5]^ As a result, GDDs are increasingly used for refractory glaucoma or as the primary procedure.

One of the most frequently implanted GDDs is the Baerveldt implant (Advanced Medical Optics, Santa Ana, California, USA). In contrast to the Ahmed glaucoma implant (New World Medical Inc, Rancho Cucamonga, California, USA), another common device, the Baerveldt implant does not have a flow restrictor and requires a ligature suture that ties off the lumen to prevent hypotony^[6]^ until a capsule has formed around the implant after four to six weeks.^[7]^ The clinical definition of hypotony is IOP low enough to result in vision loss caused, for instance by corneal edema, astigmatism, cystoid macular edema, or maculopathy.^[8]^ Since GDDs are often used in severe glaucoma with advanced optic neuropathy, a high postoperative IOP can be detrimental. To prevent this, many surgeons use spatulated needles to create slit-shaped fenestrations anterior to the ligature that permit limited flow.^[9,10,11]^ However, titrating this is challenging and the effect can range from no flow to frank hypotony.^[10]^

To address this problem and allow a complete tube ligature without fenestration, we combined Baerveldt device implantations with Trabectome-mediated ab interno trabeculectomy (NeoMedix, Tustin, CA, USA). We hypothesized that this would provide a moderate IOP reduction until the ligature dissolves allowing safe flow into a fully integrated Baerveldt implant. To achieve a balanced comparison of Baerveldt implants to Baerveldt implants with the same session Trabectome surgery, we used *Coarsened Exact Matching (CEM),* a computation-intense, modern statistics method^[12,13,14,15]^ that we applied to similar questions before.^[16,17]^ Although a randomized controlled trial remains the most effective tool to reduce bias and patient selection, modern statistical matching strategies can deliver a highly balanced assessment of real-world patient data in a retrospective analysis.

##  METHODS

This study was approved by the Institutional Review Board of the Human Subjects Research Committee at the University of Pittsburgh. An informed consent was not required for this retrospective comparative case series. Our research adhered to the tenets of the Declaration of Helsinki and the regulations of the Health Insurance Portability and Accountability Act. We identified patients who underwent a primary Baerveldt implantation (B) or primary Baerveldt implantation with Trabectome surgery (BT) between 2008 and 2015 using the Current Procedural Terminology (CPT) codes. All procedures were performed by four glaucoma fellowship-trained surgeons. Only patients older than 18 years with medically uncontrolled IOP were included. The exclusion criteria were neovascular glaucoma, uveitic glaucoma, and history of prior ocular surgery (except uncomplicated phacoemulsification).

We collected data including basic demographics, type of glaucoma, preoperative IOP, number of glaucoma medications, best-corrected visual acuity (BCVA), and type of surgery and complications as well as the postoperative IOP, number of medications, and BCVA.

The primary outcome measure was success defined as 5 mmHg < IOP ≤ 21 mmHg and ≥ 20% reduction of IOP from baseline at two consecutive visits, no need for further glaucoma surgery, and no loss of light perception. The secondary outcome measures were IOP, BCVA, and the number of medications. We defined hypotony as an IOP low enough to cause vision loss from corneal edema, astigmatism, cystoid macular edema, or maculopathy.^[8]^

We measured the IOP with a Goldmann applanation tonometer (GAT; Haag-Streit, Konig, Switzerland) at the first day, 1 week, 4 ±1 weeks, 2–4 months, 5–7 months, 8–10 months, and 11–13 months. If more than one visit occurred during these intervals, the visit closest to the months 6, 9, or 12 was chosen.

### Surgical Technique

In patients undergoing a Baerveldt implantation associated with Trabectome surgery, the Trabectome surgery was performed first. Briefly, the patient's head was tilted 30° away from the surgeon and the microscope was tilted in the opposite direction. A temporal 1.6 mm clear corneal incision was created. The tip of the handpiece was advanced into the anterior chamber and engaged with the nasal trabecular meshwork (TM). Ablation was initiated and advanced in anticlockwise direction for 90° followed by another clockwise 90° clockwise ablation at the original starting point. The handpiece was withdrawn from the anterior chamber, and the incision was hydrated to seal.

At the beginning of the Baerveldt implantation, a fornix-based conjunctival flap was fashioned, and the subtenon's space was dissected until enough space was created for the implant. The wings of the 350 mm*mm Baerveldt implant were inserted under the superior and lateral rectus muscles. The plate was sutured to the sclera 10 mm posterior to the limbus. The tube was cut with the bevel up to allow an intracameral length of 2 mm. The tube was ligated near the plate junction with a 7-0 polyglactin 910 suture (coated VICRYL, Ethicon, Somerville, NJ, United States) and tested with BSS to confirm water tightness. The tube was then inserted into the anterior chamber through a tunnel created with 23-gauge needle and secured to the sclera with a 7-0 polyglactin loop stitch. In group B, but not in group BT, the tube portion anterior to the ligature was fenestrated with a single pass of the spatulated 7-0 needle. The tube was covered with a scleral patch graft and the incision of conjunctiva and Tenon's layer was reapproximated. Most of the procedures were performed by NAL except****a few eyes in B group that were operated by other surgeons.

At the conclusion of the surgery, an antibiotic (moxifloxacin) and steroid (1% prednisolone acetate) drops were applied. The antibiotic was used four times per day for one week while the steroid eye drops were used four times per day for one month and then tapered by one drop application each week.

### Statistical Analysis

Demographical data were compared using the Mann–Whitney U test and Chi-squared test for continuous and categorical variables, respectively. To avoid eliminating data with missing values, *Multiple Imputation* in R was used (Core Team R (2018) R: A Language and Environment for Statistical Computing. R Foundation for Statistical Computing, Vienna, Austria). Missing values of the incomplete dataset were imputed *m* > 1 times, thus creating *m* completed datasets. Second, each of the *m* completed datasets were analyzed independently. Finally, the results from each of the *m* analysis were pooled into a final result. Missing data like age, gender, and race were imputed by generating five similar but non-identical datasets. Groups were then matched by *Coarsened Exact Matching* in R,^[18]^ based on the age, race, type of glaucoma, baseline IOP, and number of preoperative glaucoma medications.

Univariate linear regression was used to examine IOP reduction after surgery. Variables statistically significant were included in the final multivariate regression model. A *P*-value < 0.05 was considered statistically significant. Continuous variables were expressed as mean ± SD. All analyses were performed using R.

To compute the survival of subjects in each group, Kaplan–Meier survival plots were determined and compared using the log-rank test. Statistical significance was set at *P* < 0.05. Success was defined as the 5 mmHg < IOP ≤ 21 mmHg, and IOP reduction ≥ 20% from baseline, and no need to reoperation for glaucoma.

##  RESULTS

A total of 175 eyes undergoing primary glaucoma surgery (60 eyes in the BT group and 115 eyes in the B group) were enrolled in this study. Coarsened Exact Matching resulted in 51 eyes in the BT group matched to 51 eyes in the B group. All eyes had completed one year of follow-up visits. Ina addition, there was no significant difference between the two groups regarding ethnicity, IOP, the number of IOP-lowering medications, glaucoma type, and the degree of VF loss (P > 0.05, Table 1).

The mean age of the study participants was 70.7 ± 11.1 years in the BT group and 67.2 ± 15.7 years in the B group (*P* = 0.116). Thirty patients (59%) underwent phacoemulsification at the time of glaucoma surgery in each group (*P* =1.00). Primary open-angle glaucoma was the most common diagnosis in both groups (65.0% and 56.5% in BT and B groups, respectively, *P* = 0.516).

**Figure 1:**
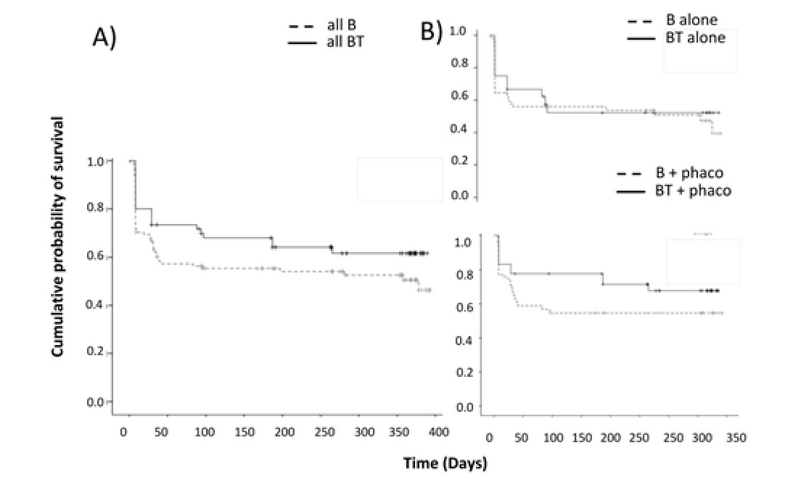
(A) Kaplan–Meier survival plots for the BT and B groups with success defined as a final IOP of ≤ 21 mmHg and a 20% reduction from baseline. Success rates of BT and B was similar in both groups. (B) survival plots of the BT and B for subgroup analysis separated by (B) glaucoma surgery alone and (C) same-session phacoemulsification (lower right).

Kaplan–Meier survival curves (Figure 1) showed a mean duration of survival of 261.9 ± 21.9 days in the BT group and 220.28 ± 17.5 in the B group with no statistically significant difference between the two groups (log rank = 2.53 *P* = 0.11). The cumulative probability of qualified success at 3, 6, and 12 months was 74%, 64%, and 61% respectively in the BT group, and 66%, 52%, and 50% in the B group.

**Figure 2:**
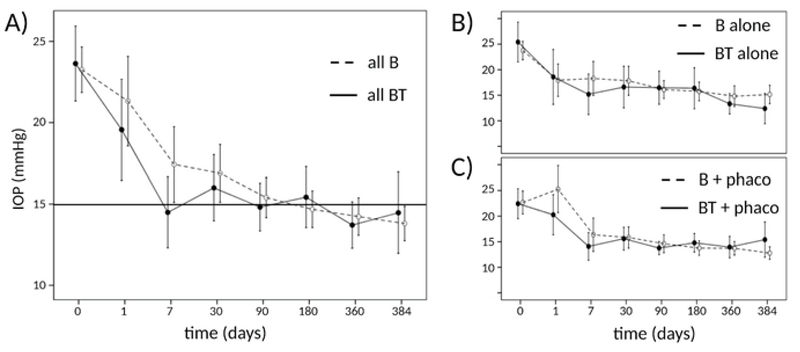
IOP in the B and BT groups. (A) IOP in the BT group was similar to the B group and trended toward a lower average although tubes in the BT group were not fenestrated and trended toward a lower average IOP. (B) B and BT techniques as stand-alone procedures. (C) B + phacoemulsification had a higher IOP on day 1 compared to subsequent IOPs. No such peak was seen in the BT group. Mean ± 95% confidence interval.

**Figure 3:**
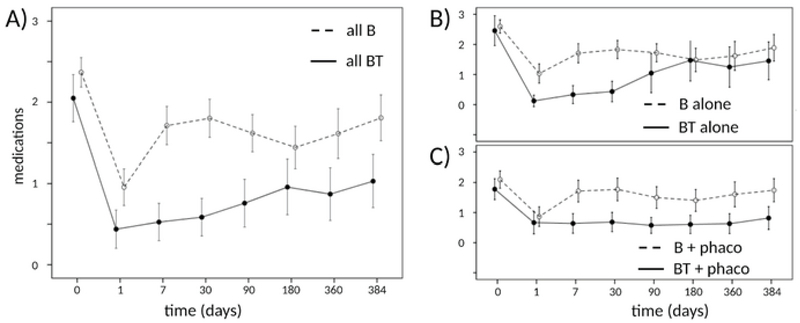
Preoperative and postoperative mean eyedrops for BT and B. Mean pre- and postoperative glaucoma medications for subgroup analysis separated by glaucoma surgery only (B) and same session phacoemulsification (C). Represented as mean ± 95% confidence intervals.

In the subgroup analysis (Table 2), the mean survival duration was 285.5 ± 25.8 days in the BT group with phacoemulsification versus 225.8 ± 25.9 days in the B group with phacoemulsification (log Rank = 2.17, *P* = 0.14). Results for glaucoma surgery alone were 221 ± 37.2 days in the BT group and 215.5 ± 23.6 days in the B group (log-rank = 0.24, *P* = 0.624, Figure 1). Survival of BT with phacoemulsification was not significantly different from B with phacoemulsification (log-rank = 1.18, *P* = 0.18). Similarly, there was no difference between the B with phacoemulsification and B alone (log-rank = 0.45, *P* = 0.50). In cases with concurrent phacoemulsification, the success rate was 56% in the BT and 52% in the B (*P* = 0.65), while in the stand-alone glaucoma surgery, the success rate was 61% in the BT and 51% in the B group (*P* = 0.31).

IOP was significantly decreased from 23.5 ± 2.4 mmHg at baseline to 14.1 ± 2.7 mmHg at final follow-up in the BT group (*P* = 0.001, Figure 2). The corresponding values for the B group were 23.2 ± 2.0 mmHg and 13.9 ± 1.6 mmHg, respectively (*P* = 0.001). IOP varied more in the B group than in the BT group during the early postoperative phase with 6.3% of hypotony occurring in the BT versus 12.8% hypotony detecting in the B group. During the one-year follow-up, 7 (13.7%) patients in the BT group and 18 (35.2%) in the B group experienced hypotony (*P* =0.04). Most of the hypotony episodes were within the first month before suture opening.

There was no significant difference in IOP at the final follow-up (*P* = 0.98). Eyes in the BT group experienced a 9 ± 9.1 mmHg reduction in IOP within one week after the surgery, compared to a 6 ± 12.3 mmHg IOP reduction in the B group (*P* = 0.09). On day one, IOP was comparable between BT with and BT without phacoemulsification (20.3 ± 11.1 mmHg versus 18.6 ± 12.7 mmHg, *P* = 0.56). The B group with phacoemulsification had a significantly higher IOP on day 1 compared to the B group without phacoemulsification (23.2 ± 14.3 mmHg versus 17.9 ± 11.4 mmHg, *P* = 0.041).

The baseline number of glaucoma medications was 2.3 ± 0.3 in both groups (Figure 3). However, the BT group needed significantly fewer drops at all postoperative visits. At the final follow-up visit, the number of glaucoma medications was 1.1 ± 0.3 drops in the BT and 2.0 ± 0.4 drops in the B (*P* = 0.003, Figure 3) groups.

The mean BCVA at the baseline was 0.64 ± 0.85 logMAR in the BT and 0.55 ± 0.75 logMAR in the B (*P *= 0.663) groups. Corresponding numbers for the final follow-up visit were 0.72 ± 1.07 and 0.63 ± 0.97 logMAR, respectively (*P* = 0.668).

The most common observation after the surgery was hyphema in 30% of the eyes in the BT group and 10% in the B group. Two eyes in the BT group and three eyes in the B group had shallow anterior chamber early after surgery which resolved spontaneously. There was one case of choroidal detachment in each group that responded to conservative management.

##  DISCUSSION

Both BT and B techniques were effective in reducing IOP. The IOP reduction of 31% at the one-year follow-up was comparable to previous reports.^[19,20,21]^ However, the success rate in our study was lower than the reported range in the literature;^[19,20,21]^ most likely the results of a lower baseline IOP in our study as compared to others. Although there was a trend toward greater IOP reduction following BT technique compared to B technique, a statistical significance was not reached. However, the BT group required significantly fewer medications postoperatively. The number of glaucoma medications at one-month postoperative visit was nearly three times as high in the B group as surgeons struggled to control the pressure in this phase of bleb maturation.

Early complications of GDDs included hyphema, a shallow or flat anterior chamber, tube-corneal touch, corneal edema, and suprachoroidal effusion.^[6,22]^ These complications are induced by postoperative hypotony, more common in the non-valved devices when the flow is not restricted and when fenestration of the tube yields excessive flow.^[23]^ Complete ligation of the tube can prevent postoperative hypotony,^[24,25,26,27]^ but high postoperative IOPs can be dangerous to eyes with advanced glaucoma damage. Therefore, tube ligation is often carried out in conjunction with intraoperative longitudinally oriented, 2 mm fenestrations proximal to the ligation.^[23]^ Despite this, a postoperative hypertensive spike may develop secondary to obstruction of the fenestration, slit malfunction, or an insufficient number of fenestrations.^[9,28]^ It is hard to titrate the function of these fenestrations and they are hardly reproducible. Intraoperative techniques such as irrigating the tube after fenestration, cautery of the bleeding episcleral vessels, and removing the debris from the field of surgery are proposed to improve the outcomes of fenestrations but it is still unpredictable. Considering these limitations, we added the same session Trabectome surgery to prevent postoperative IOP spikes. This procedure enhances the outflow by plasma-mediated ablation of the TM and has a long track record of efficacy and safety in various types of glaucoma.^[29,30,31,32,33]^

Although Trabectome is a microsurgical glaucoma surgery and is often used in earlier glaucoma stages,^[33]^ recent studies suggest it can be effective in more severe glaucoma.^[34]^ While the success rate of Trabectome after failed trabeculectomy and tube shunt procedure supports its role in the management of severe glaucoma,^[35,36]^ many eyes at that stage cannot afford a surgical failure, that can occur if the conventional drainage system downstream of the TM also has a reduced flow capacity. The effect of trabecular ablation is immediate and controls IOP until absorbable ligation sutures dissolve, and the Baerveldt implant begins to function. In contrast to the temporary effect of fenestrations, the IOP lowering of Trabectome persists after the ligature suture is absorbed and has the additive effect of reducing glaucoma medications. This is not a small feat as nearly half of all glaucoma patients experience local and systemic side effects of topical drops.^[37]^ Adverse medication effects are an important reason for non-adherence^[38]^ and can also jeopardize the success of glaucoma surgery.^[39,40]^ Conversely, reducing eye drops measurably improves the quality of life.^[41]^

Since cataract and glaucoma frequently coexist, many individuals in both groups underwent both surgeries. IOP in BT group combined with phacoemulsification was not significantly different from BT alone, reflecting our prior results that phacoemulsification does not add to the IOP-lowering effect when combined with Trabectome surgery.^[16,17]^ Although phacoemulsification has been advocated as providing a trabeculoplasty-like, additional IOP reduction,^[42]^ glaucomatous TM is often unpredictable resulting in IOP spikes at the time of stand-alone cataract surgery^[43,44]^ as we have observed here as well on day 1 in patients in group B with phacoemulsification. The results of our study showed that this potentially dangerous IOP spike could be prevented by the same-session Trabectome surgery. Conversely, no severe hypotony was seen in the BT group which did not require tube fenestration.

Limitations of this study are inherent to the retrospective nature compared to randomized controlled trials. However, the *CEM* strategy used here reduces imbalances without discarding valid data. Although observational data is easy to collect compared to randomized experiments, how the treatments were assigned and other aspects of data generation are often ambiguous and difficult to control. *CEM* is a newer form of automatic, nonparametric matching to control the confounding influence of pretreatment control variables by achieving an acceptable balance between treated and control groups.^[18]^ Additionally, this study was conducted at a single tertiary academic center, so the results cannot easily be generalized to other practice facilities. It should be noted that Trabectome surgery is a new skill even for experienced surgeons and takes at least 30 eyes to maximize the outflow,^[44]^ therefore, a complete 180° TM removal with Trabectome may not always be achieved.

In summary, we found that Baerveldt implants with same-session Trabectome surgery resulted in a significantly decreased number of glaucoma medications and avoided both severe hypertension and hypotension, thereby negating the need for tube fenestration.

##  Financial Support and Sponsorship

The authors would like to acknowledge the support from The Initiative to Cure Glaucoma, The Eye and Ear Foundation of Pittsburgh; NIH CORE Grant P30 EY08098 to the Department of Ophthalmology; an unrestricted grant from Research to Prevent Blindness, New York, NY.

##  Conflicts of Interest

There are no conflicts of interest.

##  Funding
